# Rapid Screening of Forskolin-Type Diterpenoids of *Blumea aromatica* DC Using Ultra-High-Performance Liquid Chromatography Tandem Quadrupole Time-Of-Flight Mass Spectrometry Based on the Mass Defect Filtering Approach

**DOI:** 10.3390/molecules24173073

**Published:** 2019-08-23

**Authors:** Lili He, Zhifeng Zhang, Caiyun Yao, Jianhua Miao, Bingxiong Yan, Lingling Wu, Limei Pan, Zhijun Song, Shugen Wei

**Affiliations:** 1 National Engineering Institute for the Research and Development of Endangered Medicinal Resources in Southwest China, Guangxi Botanical Garden of Medicinal Plants, Nanning 530023, China; 2 Institute of Qinghai-Tibetan Plateau, Southwest University for Nationalities, Chengdu 610041, China

**Keywords:** *Blumea aromatica* DC, foskolin, diterpenoids, mass defect filter, UHPLC-QTOF-MS/MS

## Abstract

The discovery of new active compounds of natural products tends to be increasingly more challenging due to chemical complexity and unpredictable matrices. Forskolin is an active natural labdane-type diterpenoid ingredient widely used worldwide for the treatment of glaucoma, heart failure, hypertension, diabetes, and asthma, and is expected to be a promising anticancer, anti-inflammation, and anti-HIV agent. In recent years, demand for forskolin in the medicine market has increased dramatically. However, natural forskolin originates exclusively from traditional Indian herb medicine Coleus forskohlii (Willd.) Briq. In a previous study, we isolated a series of diterpenoids including an 8,13-epoxy-14ene labdane carbon skeleton from *Blumea aromatica* DC. In order to identify alternative plant resources, a novel and effective strategy was proposed for the screening of potential forskolin-type diterpenoids (FSKD) compounds obtained from *B. aromatica*, using the mass defect filtering (MDF) strategy via ultra-high-performance liquid chromatography tandem quadrupole time-of-flight mass spectrometry (UHPLC-QTOF/MS) approach. Within a narrow, well-defined mass defect range, the strategy developed could significantly improve the detection efficiency of selected FSKD compounds by filtering out certain major or moderate interference compounds. Additionally, the MS/MS cleavage behavior and the characteristic diagnostic ions of the FSKD compounds were proposed to be used in aiding structural identification of the filtration compounds. As a result, a total of 38 FSKD of *B. aromatica* were filtered out and tentatively identified. To the best of our knowledge, it was the first time that these forskolin-type diterpenoids were identified in *B. aromatica*, which significantly expands our understanding of the chemical constituents of *Blumea* species, and allows *B. aromatica* to be used as a potential alternative plant resource that contains these forskolin-type active compounds. The strategy proposed was proven efficient and reliable for the discovery of novel compounds of herbal extracts.

## 1. Introduction

It is well known that natural products, particularly those with novel chemical structures, are recognized as playing an important role in the discovery of drugs that are beneficial for human health. However, it is still a great challenge to explore active constituents of natural sources due to the chemical diversity and complex matrices of natural products. The traditional phytochemistry approach, which involves the extraction, isolation, complicated chemical manipulations, and spectroscopic analysis of these active constituents is time-consuming and laborious, and also tends to result in the loss of minor or unstable components during the separation process.

Recently, ultra-high-performance liquid chromatography tandem with mass spectrometry (UHPLC-MS), especially with high-resolution mass spectrometry (HRMS), has begun to play an important role in the detection and identification of complex compounds used in traditional Chinese medicine (TCM) due to its high mass accuracy and resolution [1,2]. However, the main difficulty associated with structural identification may be the process used to acquire mass complex HRMS data. In previous reports, complex components of TCM have been detected and identified mainly based on MS/MS fragmentation, which is usually inefficient and labor-intensive, since it is usually done individually using manual processes. Additionally, this approach is largely dependent on the fragmentation patterns summarized from reference compounds [3,4]. It has made structural characterization more difficult, especially due to the lack of chemical standards. Furthermore, progress has been hindered further by the large number of interfering substances contained in crude herbal extracts.

Therefore, in order to rapidly detect and efficiently filter as many signals of interest as possible, a novel and effective data processing technique, the mass defect filtering (MDF) method has been explored. In fact, the identification of target compounds from a complex matrix using the MDF strategy has been used widely in MS data processing [5,6,7].

It is well known that active components of natural herbs always share a similar core carbon skeleton, with variations, such as hydroxyls, formyls, carbonyls, acetyls, and glycosylations, or a combination of these, in their chemical substitution groups. Theoretically, core substructures with different substituents always possess only relatively minor changes in their mass defect value. The mass defect profile of the compounds that share a similar carbon skeleton structure or substructure usually change within a limited range. Therefore, when analyzing a crude extract, a majority of interfering components can be excluded automatically from the complex samples by a defined MDF value which facilitate the identification of target structural analogues in TCM [8,9,10].

Forskolin, a labdane-type diterpenoid, is an active natural compound with a unique complex structure that originates from a well-known traditional Indian medicinal herb, *Coleus forskohlii* (Willd.) Briq. [11], which is used worldwide for the treatment of glaucoma, heart failure, hypertension, diabetes, and asthma based on its activity as a cyclic AMP booster [12,13], and is a promising anticancer [14,15], anti-inflammation [16,17], and anti-HIV [18] agent. Currently, commercial production of forskolin relies mainly on the extraction from its only known natural source, *Coleus forskohlii*, which is distributed in India and Southeast Asia [11]. At present, due to the significance of its activity, the market demand for forskolin will keep growing. However, reliable and sustainable commercial forskolin extraction from *C. forskholii* will become impossible due to the dramatic decrease of its natural source and low extraction yields. Great effort has been made globally to identify more natural sources containing forskolin type active compounds, in order to meet the market demand. However, as far as we know, there are only a few plants that have been identified to contain these active compounds [19].

*Blumea aromatica* DC., a traditional Chinese medicinal plant, known as Shanfeng in Chinese, belongs to the composite family and is a perennial herb widely distributed throughout Southwest China, as well as the southern and southeastern regions of Asia. Shanfeng has been widely used as traditional Chinese medicine for the treatment of rheumatism, arthralgia, and eczema. However, only a few detailed reports are available on the chemical properties of *B. aromatica* [20]. In our previously study, a total of five diterpenoid compounds were isolated from *B. aromatica* in our lab. It was notable that the structures of the diterpenoid isolated from *B. aromatica* shared a core skeleton which was similar to that of forskolin. As far as we know, this was the first time that these forskolin-type diterpenoid (FSKD) compounds were found in *B. aromatica*.

In order to find more FSKDs and fully profile the FSKDs in *B. aromatica*, identify other alternative plant resources containing forskolin active ingredients, and understand the effective material basis and pharmacological activity of crude herbs, it is urgently required to develop a sensitive and reliable method to characterize the FSKD in *B. aromatica*. In this study, we describe an effective and facile approach for the overall capture of the FSKDs in *B. aromatica* based on UHPLC-QTOF/MS coupled with the MDF approach. For the first time, a total of 38 FSKDs were filtered out and tentatively identified from *B. aromatica*. It is expected that the Method and Results of the present study would expand our understanding of the chemical constituents of *B. aromatica*, and provide a helpful strategy to identify natural plant resources containing FSKD active components, as well as to be used as an effective tool to screen for chemical targets for future phytochemical and pharmacological activity studies on these important herbal medicines.

## 2. Results

**Figure 1:**
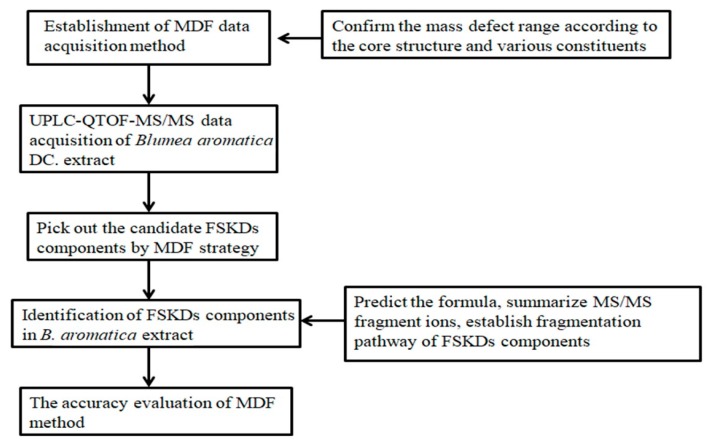
Flow diagram illustrating the approach used to screen forskolin-type diterpenoids from *B. aromatica*.

According to the proposed screening strategy below, first, the MDF filtration method was established based on the FSKDs constituents which have been reported in Coleus forskohlii [21,22]. Secondly, the extract of *B. aromatica* sample was analyzed by UHPLC-QTOF-MS/MS at both positive and negative modes. Then, the developed MDF method was performed to pick out the potential precursor ions of the FSKDs. Finally, the structures of the screened compounds were further elucidated based on MS/MS spectra and fragmentation pathways were summarized using the reference substance (see Figure 1).

The base peak ion (BPI) chromatogram of the extraction of *B. aromatica* before and after MDF filtration is shown in Figure 4. In this study, a total of 38 FSKDs of the *B. aromatica* sample were selected and characterized (Table 1).

### 2.1. Optimization of Sample Preparation and UHPLC-QTOF-MS Analysis Conditions

The extract parameters were optimized in order to obtain a higher extraction efficiency, including the following: the type of extraction method (ultrasonic and refluxing); extraction solvent (different methanol/water ratio solvent of 70:30, 80:20, and 85:15, *v/v*); and extraction time (20, 30, 45, and 60 min). The chromatograms of *B. aromatica* extract with different extraction conditions were available in Supplementary Materials (online at www.mdpi.com/xxx/s1). The results were evaluated by the number of detected peaks after UPLC-QTOF-MS analysis. In the end, ultrasonic extraction conducted for 45 min with 80% methanol (*v/v*) as a solvent was selected as the optimal extraction conditions for crude sample preparation.

The UHPLC-Q-TOF-MS conditions, such as the column type, the composition of mobile phase, ionization mode, capillary voltage, and collision energy, were also evaluated to get a shorter analytical time and satisfactory separation effect. The mobile phase additives may have a significant influence on signal response. It was found that the aqueous mobile phase containing formic acid (0.1% *v/v*) could improve the peak shape and give higher ionization efficiency, especially that of trace peaks. Thus, a gradient elution system composed of acetonitrile-0.1% aqueous formic acid was chosen as the eluting solvent system to give the optimal chromatographic peak shapes and resolution. In order to shorten analytical time and obtain satisfactory separation, two RP-C_18_ columns, Waters ACQUITY UPLC BEH C18 (100 × 2.1 mm, 1.7 μm) and ACQUITY UPLC HSS T3 (100 × 2.1 mm, 1.8 μm), flow rate (0.3 mL/min, 0.4 mL/min, 0.5 mL/min, and 0.6 mL/min) and column temperature at 30 °C, 35 °C, and 40 °C were evaluated. Finally, ACQUITY UPLC HSS T3 column (100 × 2.1 mm, 1.8 μm) with the temperature at 35 °C and flow rate of 0.5 mL/min were chosen to obtain optimal chromatographic results. MS spectra were scanned at both positive and negative modes. The positive scanning mode was selected due to its higher ionization efficiency, and higher abundance fragment ions, even at low collision energy. Meanwhile, the precursor ions prone to form the [M + HCOO]^−^ quasimolecular ions were scanned under a negative scanning mode, which helped to confirm the molecular formula. The collision energy was optimized in the range of 20–60 V to obtain better MS/MS spectra results for the FSKD. In general, low collision energy is applied to ions with a low molecular weight to produce satisfactory fragment ions. In contrast, large molecules require high collision energy to obtain more fragment ions.

### 2.2. Establishment of the MDF Filtration Method

The MDF strategy has been applied to greatly improve the efficiency of the identification of bioactive constituents in TCM [23,24,25]. The components share the same core structure but have different substituent groups, which result in relatively narrow and defined mass defect shifts. Hence, in order to follow the MDF strategy, it was essential to define the mass defect value based on the combination of core structure and substituents.

**Figure 2:**
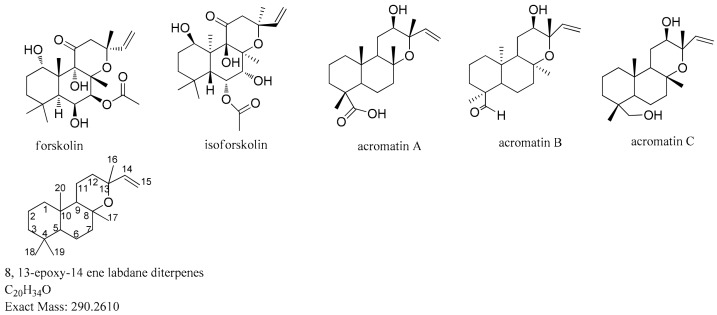
The chemical structure of reference compounds.

On the basis of the concept of MDF described above, the first step was to ensure filtering reference skeleton based on published forskolin analogues [19,21,22]. The next step was to establish the mass defect range based on the core structure and substituent group combination. Then, filtration was applied to the chromatogram based on the setting expressed as central formula ± mass defect tolerance. Finally, background ions were automatically removed, and characteristic ions remained visible after screening. In this study, the filtering parameters were established using the “accurate mass filter” function in MassLynx 4.1 software (Waters Corp., Milford, MA, USA). On the basis of the information published on these compounds, the filter reference was determined as 8, 13-epoxy-14 ene labdane diterpenes (C_20_H_34_O) and is illustrated in Figure 2.

**Figure 3:**
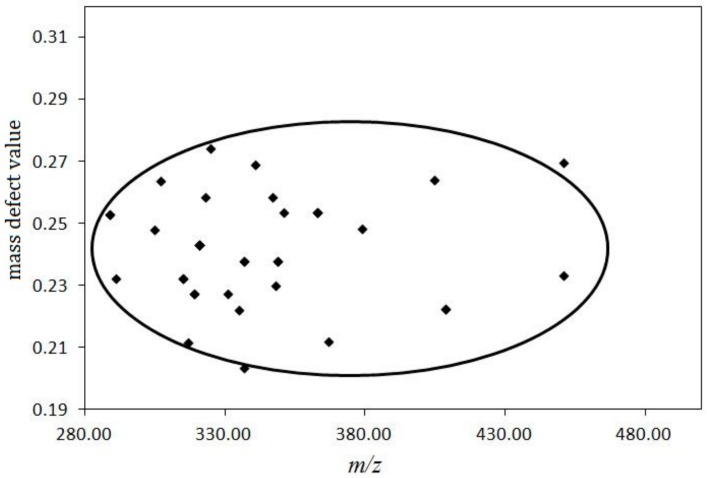
Mass defect filtering value distribution of 8, 13-epoxy-14 ene labdane type diterpenes.

The substituents of the core structure mainly included carbonyl-function at the C-11 position, hydroxylation at C-1, C-6, C-7, and C-12 positions, acetylation or deacetylation, and other oxidized derivatives. The mass defect values were different between various substituents, with both carbonylation and acetylation increasing the mass defect value while hydroxylation decreased the mass defect value. On the basis of extract mass summaries of published forskolin analogues and mass defect value of the various substituents, the exact mass value was set on the X-axis and the mass defect value on the Y-axis. The mass defect distribution diagram obtained is shown in Figure 3. It is interesting to note that all the components were found to be clustered in an elliptical range. Thus, the calculated minimum and maximum mass defect values of the labdane-type diterpenoids were found to be in the range of 0.2171–0.2788 Da, while the mass ranged from 280–460 Da. Therefore, the filtering template used for FSKD screening was set as follows: mass range 280–460 Da, MDF value set at 0.25 Da, with an upregulation of 30 mDa and downregulation of 40 mDa. The mass error of the predicted chemical formula should be less than 10.00 ppm, while the element composition was set at C (18–25), H (28–40), and O (2–7).

### 2.3. FSKD Analogue Identification After Filtration Using the MDF Approach

**Figure 4:**
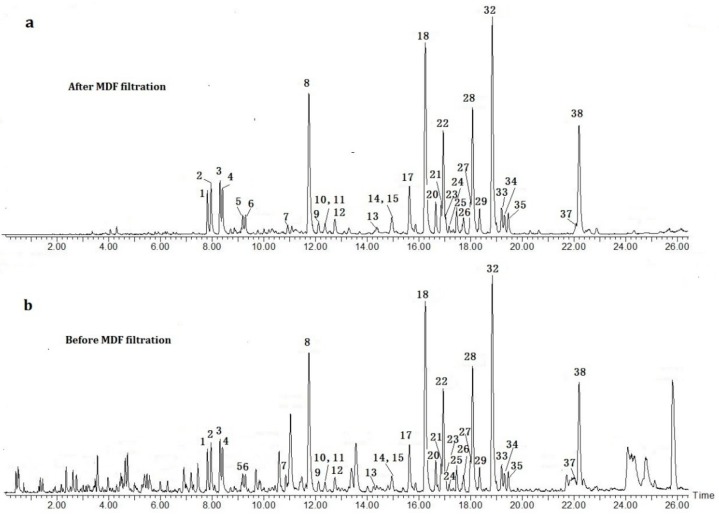
The ultra-high-performance liquid chromatography tandem quadrupole time-of-flight mass spectrometry (UHPLC-QTOF-MS) base peak intensity (BPI) chromatograms of *B. aromatica.*: (**a**) After MDF filtration and (**b**) Before MDF filtration.

After filtration using the optimized MDF approach, all interfering ions with mass defects that were significantly not within the filter window were excluded. The base peak intensity (BPI) chromatograms before and after filtration are shown in Figure 4. The noise level of the filtered chromatogram was much lower than that of the chromatogram without MDF filtration. As a result, a total of 38 peaks of the potential FSKD were filtered out within 28 min of chromatographic running time (Table 1). Similar types of chemical structures always possess similar MS/MS fragmentation behaviors and lead to common characteristic ions [8,9,23]. The fragment ions of each filtered peak are listed on Table 1. The accurate mass and element composition of the precursor ions and fragment ions were calculated using the tool of “elemental composition” of MassLynx V 4.1 software. The mass error of the predicted chemical formula of less than 10.00 ppm were selected as candidate compounds. Meanwhile, MassFragmentTM software was used to assist with the elucidation of chemical structure based on a scoring system. The structural identification of the FSKDs were further confirmed using reference compounds, published literature, the MS/MS fragmentation pathway, key product ions filtration, SciFinder, ChemSpider, and other online databases. All related data of the identified components are summarized in Table 1.

The cleavage pathways of FSKDs of *B. aromatica* are proposed based on MS/MS data of the reference compounds. In previous research, diterpene compounds with a similar skeleton were found in *B. aromatica*, of which the structure belongs to 8,13-epoxy-14 ene labdane type diterpenes, and it is similar to the structure of forskolin. Until now, five FSKDs have been isolated from *B. aromatica* by our group. Three of them, acromatin A, acromatin B and acromatin C, together with another two commercial standard compounds, forskolin and isoforskolin, were employed as template structures to study the fragmentation patterns of the FSKDs, which can subsequently enhance the comprehensive identification of other FSKDs in *B. aromatica* with the same core skeleton.

#### 2.3.1. Structural Elucidation of Acromatin A, Acromatin B, and Acromatin C

On the basis of previous studies, compounds acromatin A, acromatin B, and acromatin C extracted from *B. aromatica* were isolated and characterized by our team and were identified using UV, IR, HRMS, and NMR. Their chemical structures are shown in Figure 2. The structures of acromatin A, acromatin B, and acromatin C were found to share the same core skeleton of 8,13-epoxy-14 ene labdane diterpenes, with only differences in chemical configurations of the substituent group at the C-4 position, for which the substitutions groups were carboxyl, aldehyde, and methylene hydroxyl for acromatin A, acromatin B, and acromatin C, respectively. Unlike forskolin, no carbonyl group was substituted at the C-11 position and no acetyl group substitution at the C-6 and C-7 positions was found in these FSKDs extracted from *B. aromatica*. Their main fragmentation pathway was the cleavage of COOH, CHO, and CH_2_OH at C-4 and the neutral losses of H_2_O groups, along with the cleavage of the C_9_-C_11_ and C_8_-O-C_13_ bonds of the C-ring.

As regards to acromatin C, the deprotonated molecular ion (*m/z* 335.2226, [M − H]^−^) was observed at t_R_ = 18.08 min under negative ionization mode and adductive ion *m/z* 690.4946 [2M + H_2_O]^+^ under the positive mode. The elemental composition of the quasimolecular ions were calculated to be C_20_H_32_O_4_ using the “elemental composition” of MassLynx v4.1 software. It is notable that the SFKDs in *B. aromatica* are prone to form stable precursor ions [M − H]^−^ or [M + COOH]^−^ under the negative scanning mode, however, it is difficult to break these down to obtain fragment ions even under high collision energy. In contrast, the fragmentation ions easily formed under the positive ionization mode, even at low collision energy (6 V). Therefore, in our study, both negative and positive scanning modes were used to obtain more information for structural elucidation of SFKDs in *B. aromatica*.

**Figure 5:**
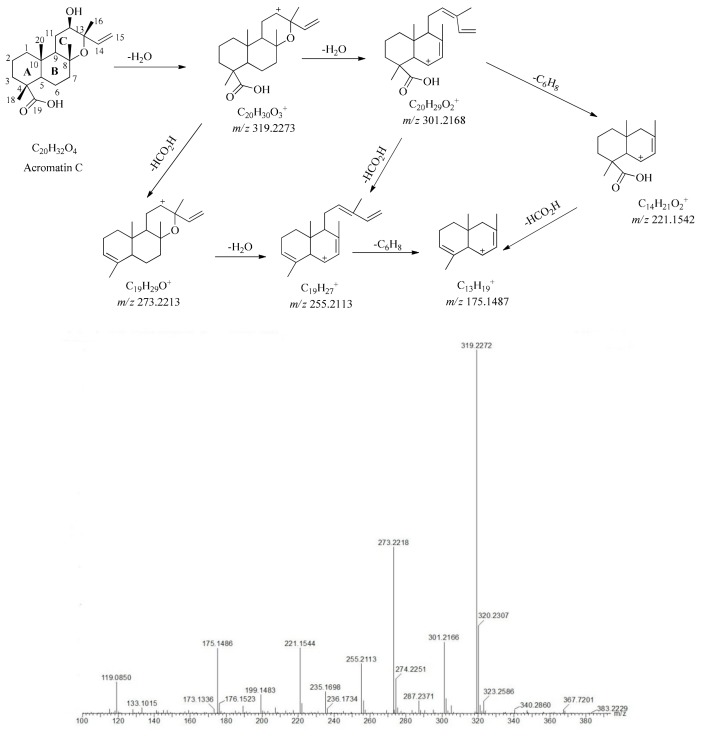
Proposed cleavage pathway and the MS/MS spectra of acromatin C.

As shown in Figure 5, under positive ionization mode, the main product ions at *m/z* 319.2274 [M − H_2_O]^−^ and 301.2170 [M − 2H_2_O]^−^ corresponded to the successive neutral loss of H_2_O. There was a 46 Da difference between the fragment ion at *m/z* 319.2274 and 273.2220, indicating the presence of a carboxyl group. The product ion at *m/z* 255.2115 was produced by cleavage of the ion at *m/z* 273.2220 at the C_8_-O-C_13_ bond and the loss of one H_2_O group, while the product ion at *m/z* 175.1485 was produced by cleavage of the C_9_-C_11_ bond. Additionally, fragment ions at *m/z* 255.2115, 235.1695, and 221.1543 corresponded to the successive loss of the formic acid group, the cleavage of C_9_-C_11_, and C_11_-C_12_, respectively. As for the low mass fragments, such as that at *m/z* 145.1005 (C_11_H_13_) and *m/z* 133.1010 (C_10_H_13_), they were derived from the A-ring and B-ring segments. In brief, the proposed fragmentation pathways of the SFKDs were mainly derived from the neutral losses of COOH, CHO, and CH_2_OH at C-4 and H_2_O, the cleavage of C_8_-O-C_13_, and then the cleavage of A and B rings. Notably, ions at *m/z* 273, 255, 221, and 175 were found to be the characteristic fragment ions of these types of SFKDs. The prominent fragmentation pathway proposed for acromatin C is shown in Figure 4, which is expected to facilitate the characterization of SFKDs after filtration using the MDF method.

**Figure 6:**
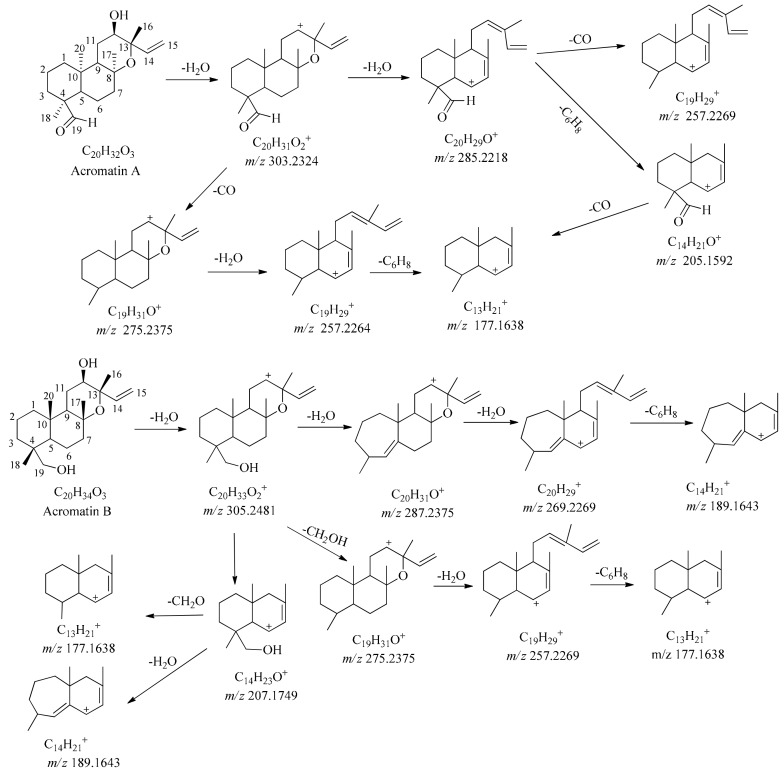
Proposed cleavage pathway and the MS/MS spectra of acromatin A and acromatin B.

The compounds acromatin A and acromatin B share the same core frame structure with acromatin C**,** with differences in the substituent group at the C-4 position. Similar to acromatin C, the cleavage pathways of acromatin A and acromatin B mainly include the neutral losses of H_2_O, CO, and CH_2_O, and the cleavage of the C_8_-O-C_13_ bond in the C-ring. Fragmentation is always triggered by rearrangement of the A-ring, resulting in the formation of a seven-member ring when the C-4 position is substituted by a -CH_2_OH group. As shown in Figure 6, acromatin B (t_R_ = 20.17 min) produces the quasimolecular ion at *m/z* 658.5023 [2M + H_2_O]^+^ and 663.4627 [2M + Na]^+^ under positive ionization mode, with a molecular formula of C_20_H_34_O_3_. Fragment ions *m/z* 305.2476 and 287.2370 are derived from the successive loss of H_2_O, accompanied by the rearrangement of the A-ring to form a seven-member ring, followed by the cleavage of the C_8_-O-C_13_ bond forming the product ions at *m/z* 269.2266 and 189.1643. Another cleavage pathway, with the neutral losses of -CH_2_OH directly without rearrangement of the A-ring, yields the product ions of *m/z* 275.2375 and further fragmentation of *m/z* 275.2368 produces ions *m/z* 257.2271 and 177.1639 due to the cleavage of C_8_-O-C_13_ bond of the C-ring (see Figure 6). The characteristic fragments for this type of SFKDs were also observed at *m/z* 275, 257, and 177, which could be used for the identification of ions in this type of FSKD structure.

#### 2.3.2. Structural Elucidation of Forskolin

**Figure 7:**
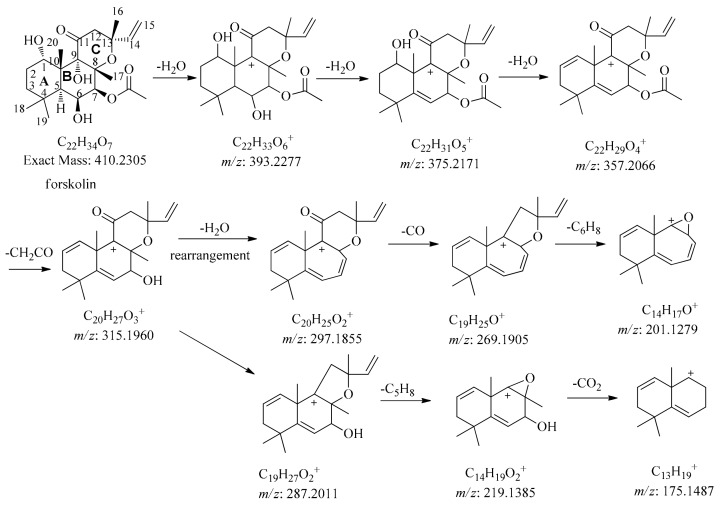
Proposed fragmentation pathway of forskolin.

Forskolin shares a common core frame structure with acromatin A, acromatin B, and acromatin C, which belong to the 8,13-epoxy-14ene labdane type of diterpenes. The differences are in the substituent group of the structure. There is usually a carbonyl substitution at the C-11 position of forskolin, dimethyl substitution at C-4, and acetyl substitution at the C-6 or C-7 position. Additionally, no hydroxyl group is substituted in the C-12 position (Figure 2). As for forskolin, the quasimolecular ion at *m/z* 411.2383 [M + H]^+^, with a molecular formula of C_22_H_34_O_7_, produced the predominant fragment ion at *m/z* 393.2272 [M + H − H_2_O]^+^, 375.2171 [M + H − 2H_2_O]^+^, 357.2066 [M + H − 3H_2_O]^+^, and 315.1960 [M + H − 3H_2_O − CH_2_CO]^+,^ were subjected to the successive cleavages of hydroxyl (-18), acetyl (-42) groups, respectively (Figure 7). Rearrangement was also observed in the cleavage pathway of forskolin.

Elimination of –OH leads to the formation of carbocation at the C-9 position, which is prone to rearrangement of the methyl group at C-17 to form a seven-member ring. The product ions at *m/z* 297.1845 [M + H − 4H_2_O − CH_2_CO]^+^, 269.1905 [M + H − 4H_2_O − CH_2_CO − CO]^+^, and 201.1276 [M + H − 4H_2_O − CH_2_CO CO − C_5_H_8_]^+^ are attributed to the neutral loss of H_2_O, CH_2_CO, and CO, respectively, after rearrangement. The abundant fragments at *m/z* 297 and 201 are the characteristic ions attributed to this type of labdane diterpenes. At the same time, the neutral loss of an acetyl group (-42) is also characteristic of the cleavage ascribed to this type of FSKDs.

### 2.4. Accuracy Evaluation of the MDF Filtration Method

The effectiveness of the MDF approach using the core substructure and substitution groups as a filter reference has been well demonstrated in the *B. aromatica* extract. After removal of the interfering compounds in *B. aromatica* extract using the above UHPLC-QTOF-MS and MDF filtration approach, the background noise of the BPI chromatograph (Figure 4) was significantly reduced and detection sensitivity was successfully enhanced. The efficiency of the MDF approach was also evaluated based on the number of FSKD compounds identified using UHPLC-QTOF-MS/MS platform divided by the number of compounds selected by MDF as potential FKSDs. Theoretically, all ions with a mass defect value within the setting range should be selected for further structural elucidation. However, some of these ions were not identified due to a weak response intensity, leading to the lack of MS/MS data and unavailability of fragment ions of the selected precursor ions under the MS conditions selected. In order to increase screening efficiency, these ions need to have a MS response intensity above 2.0 e^4^ (positive mode) or 6.0 e^4^ (negative mode) to be suitable for use in further research.

After filtering the FSKDs in *B. aromatica* extract using MDF, a total of 45 peaks were selected within the defined range. Then, UHPLC-QTOF-MS/MS was applied to characterize both target and non-target components in *B. aromatica* extract. Finally, a total of 38 potential FSKD precursor ions were selected based on accurate mass, fragment behaviors, and diagnostic ions, described above. All identified FSKDs were confirmed to be distributed in the established mass defect range. The MS/MS fragmentation patterns of the 38 FSKDs are summarized in Table 1. The BPI chromatogram of the FSKDs under the positive ion mode is shown in Figure 4. On the basis of the results of structural characterization, the accuracy of the MDF filtration method for FSKDs in *B. aromatica* was found to be 84.44%.

It is notable that, many FSKD isomers were detected in the *B. aromatica* extract. These include peaks at 14, 17, 20, 23, 25, and 29, the adduct ions [M + HCOO]^−^ at *m/z* 381.2268 and the deprotonated molecule ions [M − H]^−^ at *m/z* 335.2226, which give the same molecular formula of C_20_H_32_O_4_, as with that of acromatin C. Meanwhile, the characteristic fragment ions at *m/z* 319, 301, 273, 255, 221, and 175 are consistent with that of acromatin C. Therefore, compound 14, 17, 20, 23, 25 and 29 are suggested to be isomers of acromatin C, which are FSKD analogues. The existence of diagnostic ions at *m/z* 273.2225, 255.2115, 221.1545, and 175.1496 indicate carbonyl group substitution at the C-4 position, similar to that of acromatin C.

Peaks 9, 10, 12 and 13 give rise to deprotonated molecule ions [M − H]^−^ at *m/z* 353.23, with the same molecular formula as C_20_H_34_O_5_, indicating the isomer structure of each other. Characteristically, compounds 9, 10, 12, and 13 yield the product ions at *m/z* 337, 319, 301, 289, 273, 255, 221, and 175, which indicates that these compounds share the same or similar substructure. At the same time, the diagnostic fragment ions of 273, 255, 221, and 175 are consistent with that of acromatin C. Therefore, compound 9, 10, 12, and 13 were deduced to be FSKD analogues. Using the same strategy, other diterpenoids in *B. aromatica* extract were characterized as FSKDs.

It is noteworthy that the diagnostic fragment ions at *m/z* 297 and 201 were not detected in the filtered peaks and neither was the characteristic neutral loss of acetyl (-42) observed. Therefore, it was assumed, that they are different from the structure of forskolin, with no carbonyl group substituted at the C-11 position and no acetyl group substitution at C-6 and C-7 positions in these types of FSKDs detected in *B. aromatica* extract.

## 3. Materials and Methods

### 3.1. Chemicals and Materials

The authentic standard of forskolin and isoforskolin were purchased from the National Institutes for Food and Drug Control (Beijing, China). Acromatin A, acromatin B and acromatin C were isolated from *B. aromatica* in our laboratory and their structures were identified using ESI–MS and NMR. The purity was more than 98% (determined by HPLC). Acetonitrile (HPLC-grade) and formic acid with a purity of >98% were purchased from Merck KGaA (Darmstadt, Germany), and ultrapure water purified with a Milli-Q Academic system (Millipore, Bedford, MA, USA) was used for the LC/MS analysis. The structures of the reference compounds are summarized in Figure 2.

The herbal material was collected from Fengshan county, Hechi, Guangxi Province of China and authenticated by S. X. Feng as *Blumea aromatica DC*. and the voucher specimens (No. 153603) were deposited at the Herbarium Centre, Guangxi Botanical Garden of Medicinal Plants, Nanning, China.

### 3.2. Preparation of Sample and Standard Solutions

The air-dried sample of *B. aromatica* was pulverized into a powder and sieved through an 80-mesh screen. An aliquot of 0.5 g of dried *B. aromatica* powder was suspended in 20 mL of 80% methanol (*v/v*) in a 25 mL volumetric flask and sonicated at 100 Hz for 45 min. After reaching room temperature, the suspension was made up to 25 mL by adding 80% methanol (*v/v*). The solutions obtained were centrifuged at 13,000 rpm for 10 min, then filtered through 0.22 μm filter units, before being injected into UHPLC-Q-TOF/MS systems.

Stock solutions: A specified amount of forskolin, isoforskolin, acromatin A, acromatin B, and acromatin C were dissolved in 80% methanol-water (*v/v*) to obtain initial stock solutions at a concentration of 1.0 mg/mL, and then stored at under 4 °C in a refrigerator. All solutions were filtered using a 0.22 μm PTFE syringe filter before being injected for LC/MS analysis.

### 3.3. UHPLC-QTOF/MS Conditions

A Waters ACQUITY UHPLC I-Class system (Waters Corporation, Milford, MA, USA) was used to carry out the chromatographic separations. The analysis gradient elution with 0.1% (*v/v*) aqueous formic acid (A) and acetonitrile (B) was used as the mobile phase at a flow rate of 0.5 mL/min. The optimized gradient elution program was: 0–2.5 min, 5–10% B; 2.5–4.5 min, 10–22% B; 4.5–7.0 min, 22–28% B; 7.0–14.0 min, 28–55% B; 14.0–20.0min, 55–60% B; 20.0–22.0 min, 60–70% B; 22.0–25.0 min, 70–95% B; and 25.1–28.0 min, 5% B. Chromatographic separation was achieved with an ACQUITY HSS T3 column (100 mm × 2.1 mm, 1.8 μm; Waters, USA) at 35 ℃. The sample injection volume was set at 2 μL.

MS/MS analysis was performed on a high-resolution quadrupole time-of-flight (QTOF-MS) system (Xevo G2-S, Waters Corporation, Manchester, UK), with a performance of resolution >30,000, mass accuracy <1 ppm, and coupled with an electrospray ionization (ESI) interface. All samples were determined at both positive and negative ionization mode with mass ranging from 100 to 1200 Da over a 28 min analysis time. LockSpray™ was used to ensure MS data accuracy and reproducibility. The leucine-enkephalin (Sigma-Aldrich, Saint Quentin Fallavier, France) was used as the lock reference mass, generating [M − H]^−^ and [M + H]^+^ at *m/z* 554.2615 and *m/z* 556.2771 at the negative and positive electrospray ionization mode, respectively, with a concentration of 400 ng/mL and flow rate of 5 μL/min during MS analysis to ensure accuracy during MS analysis. The lockspray scan time was set as 0.1 s and the interval was set to 20 s.

The other QTOF-MS operating parameters were optimized as follows: nitrogen was used as the source of gas, while ultrahigh purity argon was used as the collision gas for collision induced dissociation (CID). The desolvation gas flow rate was set at 800 L/h with a temperature of 350 °C, and the cone gas was set at 20 L/h and the cone voltage was set at 40 V, the source temperature was set at 120 °C and the capillary voltage was set at 2.7 kV and 3.0 kV at the negative and positive scanning modes, respectively. For the MS/MS experiments, an MS^E^ experiment with a dynamic collision energy range of 30–50 V was conducted to obtain both precursor ions and fragment ions using one injection of the sample. The MS^E^ parameters were set as follows: function 1: *m/z* 100–1200, 0.2 s scan time, 0.02 s inter-scan delay, 40 V sample cone, low collision energy set at 6 V; function 2: *m/z* 50–1200, 0.2 s scan time, 0.02 s inter-scan delay, 40 V sample cone, 30-50 V ramp high collision energy.

### 3.4. Data Process

The MDF method was followed using the “accurate mass filter” function of Masslynx 4.1 software. The molecular formulas were obtained using the “elemental composition” of Masslynx 4.1 software. The mass error of the predicted chemical formula lower than 10.00 ppm was selected as the candidate compound. Meanwhile, MassFragmentTM software, which can automate structural assignment based on a particular scoring system, was applied to assist the elucidation of chemical structure and to ensure the accuracy and efficiency of MDF. The MS/MS fragment analysis using MassFragment^TM^ was performed at the positive scanning mode. The key parameters were set as follows: maximum mass error, double-bond equivalent DBE 0–10; electron count, odd; maximum H deficit, 6; and fragment number of bonds, 4.

## 4. Conclusions

In the present study, a powerful strategy was used to rapidly obtain the characteristic forskolin analogues of *B. aromatica* using a method of UHPLC-QTOF/MS coupled with MDF data filtration approach. Compared with the conventional method, the MDF strategy enables a majority of the interfering ions to be automatically filtered, allowing the original data to be analyzed more effectively, accurately, and rapidly. It could be used for the rapid analysis of other homologous families used in TCM, especially when there is a lack of literature and inaccessibility of standards of authentication.

A total of 38 FSKD precursor ions were characterized, or tentatively identified, in *B. aromatica* extract, for the first time. The newly discovered FSKDs significantly expand our understanding of the chemical constituents of *B. aromatica*, which is a potential alternative plant resource that contains forskolin-type diterpenoid active compounds. Accurate structural identification of chemical compounds in TCM requires integration of NMR, IR, UV, and MS data. The current DMF filtration strategy based on MS data is able to provide structural information of interested compounds used in TCM. Further research will be conducted at our lab to target the phytochemical and pharmacological activity of the FSKDs of important herbal medicines.

## Figures and Tables

**Table 1:** Foskolin-type diterpenoids of *B. aromatica* screened using the MDF method.

Peak No.	Retention Time (min)	ESI^−^, m/z *		ESI^+^, m/z	Formula	MS/MS Fragment Ions in Positive Mode **
		[M − H]^−^	[M + COOH]^−^			
1	7.81	369.2282	415.2367	388.2722 [M + H_2_O]^+^	C_20_H_34_O_6_	353.2346, 335.2242, 317.2135, 305.2133, 299.2022, 289.2181, 287.2037, 275.2031, 271.2077, 259.2080, 253.1980, 237.1858, 229.1974, 221.1555
				393.2276 [M + Na]^+^		
				741.4863 [2M + H]^+^		
2	7.95	369.2274	415.2335	393.2271 [M + Na]^+^	C_20_H_34_O_6_	353.2341, 335.2237, 317.2133, 299.2029, 289.2183, 287.2025, 275.2028, 271.2073, 259.2076, 255.2122, 253.1950, 221.1556
				741.4832 [2M + H]^+^		
				763.4637 [2M + Na]^+^		
3	8.30	369.2273	415.2336	388.2710 [M + H_2_O]^+^	C_20_H_34_O_6_	353.2343, 335.2233, 317.2122, 305.2129, 299.2018, 289.2175, 287.2021, 275.2022, 271.2075, 259.2068, 253.1958, 229.1965, 221.1552
				393.2259 [M + Na]^+^		
				741.4811 [2M + H]^+^		
4	8.40	369.2279	415.2338	388.2710 [M + H_2_O]^+^	C_20_H_34_O_6_	353.2345, 335.2234, 317.2120, 305.2132, 299.2014, 289.2176, 287.2024, 275.2027, 271.2075, 259.2065, 253.1952, 229.1965, 221.1556
				393.2260 [M + Na]^+^		
				741.4801 [2M + H]^+^		
				763.4626 [2M + Na]^+^		
5	9.19	351.2180	397.2241	370.2602 [M + H_2_O]^+^	C_20_H_32_O_5_	335.2232, 317.2122, 305.2121, 299.2022, 289.2179, 287.2014, 275.2006, 271.2074, 255.2113, 253.1974, 221.1557, 175.1492
				375.2152 [M + Na]^+^		
				705.4606 [2M + H]^+^		
				727.4465 [2M + Na]^+^		
6	9.29	351.2178	397.2248	370.2602 [M + H_2_O]^+^	C_20_H_32_O_5_	335.2232, 317.2122, 305.2121, 303.2323, 299.2022, 289.2179, 287.2021, 275.2006, 271.2074, 259.2070, 253.1974, 221.1557, 175.1491
				375.2159 [M + Na]^+^		
				705.4611 [2M + H]^+^		
				727.4465 [2M + Na]^+^		
7	10.93	351.2176	397.2245	370.2604 [M + H_2_O]^+^	C_20_H_32_O_5_	335.2235, 317.2125, 305.2120, 303.2328, 299.2020, 289.2176, 275.2010, 271.2071, 259.2073, 253.1970, 221.1553
				375.2155 [M + Na]^+^		
				705.4610 [2M + H]^+^		
				727.4466 [2M + Na]^+^		
8	11.74	307.1905	353.1953	309.2070 [M + H]^+^	C_18_H_28_O_4_	291.1969, 273.1863, 263.2017, 255.1757, 245.1913, 227.1808, 221.1551
				326.2342 [M + H_2_O]^+^		
				331.1891 [M + Na]^+^		
9	12.12	353.233	399.2389	377.2291 [M + Na]^+^	C_20_H_34_O_5_	337.2320, 319.2272, 301.2164, 289.2163, 273.2215, 271.2060, 253.1949, 221.1548, 175.1482
10	12.36	353.233	399.2389	---	C_20_H_34_O_5_	337.2322, 319.2274, 301.2160, 289.2165, 273.2210, 271.2061, 253.1946, 221.1545, 175.1484
11	12.38	365.1948	411.2001	---	C_20_H_30_O_6_	349.2005, 319.2261, 301.2149, 273.2208, 255.2085, 221.1521, 175.1489
12	12.76	353.2316	399.2379	---	C_20_H_34_O_5_	337.2365, 319.2250, 301.2152, 289.2156, 279.1592, 273.2215, 259.2047, 255.2110, 253.1702, 221.1533, 175.1490
13	14.36	353.2319	399.2379	377.2271 [M + Na]^+^	C_20_H_34_O_5_	337.2360, 319.2256, 301.2150, 289.2159, 279.1590, 273.2212, 259.2053, 255.2111, 253.1703, 221.1535, 175.1488
14	14.96	335.2217	381.2268	---	C_20_H_32_O_4_	319.2276, 301.2173, 287.2014, 289.2154, 273.2222, 269.1906, 255.2115, 221.1545
15	14.98	349.2011	395.2065	---	C_20_H_30_O_5_	333.2071, 315.1973, 287.2030, 269.1908, 255.2138, 227.1819, 221.1565
16	15.28	---	---	634.5107 [2M + H_2_O]^+^	C_19_H_32_O_3_	291.2344, 273.2238, 255.2130, 207.1768, 199.1505, 191.1453, 175.1503
17	15.63	335.2231	381.2283	---	C_20_H_32_O_4_	319.2298, 301.2192, 283.2084, 273.2283, 255.2133, 221.1560, 207.1770, 189.1659, 175.1502
18	16.25	333.2078	379.2132	335.2216 [M + H]^+^	C_20_H_30_O_4_	317.2112, 299.2007, 289.2163, 271.2057, 253.1952, 243.2113, 221.1542, 175.1483, 161.1327, 145.1014
				357.2035 [M + Na]^+^		
				331.2238 [M + Na]^+^		
				639.4576 [2M + Na]^+^		
19	16.43	---	---	331.2238 [M + Na]^+^	C_19_H_32_O_3_	291.2314, 273.2210, 255.2104, 207.1739, 199.1477, 191.1429, 175.1480
				639.4576 [2M + Na]^+^		
20	16.65	335.2207	381.2254	359.2176 [M + Na]^+^	C_20_H_32_O_4_	319.2257, 301.2152, 283.2047, 273.2204, 255.2104, 221.1533
21#	16.87	321.2061	367.2113	---	C_19_H_30_O_4_	305.2112, 287.2007, 259.2059, 241.1946, 231.2101, 221.1540, 175.1487
22	16.94	333.2064	379.2114	335.2225 [M + H]^+^	C_20_H_30_O_4_	317.2118, 303.2327, 299.2014, 285.2218, 271.2065, 253.1960, 221.1547, 207.1751, 175.1487
				357.2048 [M + Na]^+^		
23	17.03	335.2215	381.2254	337.2387 [M + H]^+^	C_20_H_32_O_4_	319.2274, 301.2170, 289.2174, 273.2222, 255.2122, 221.1542, 193.1231, 175.1484
				673.4677 [2M + H]^+^		
24#	17.16	349.2016	395.2074	---	C_20_H_30_O_5_	333.2086, 315.1972, 287.2021, 269.1918, 247.1706, 243.2122, 235.1702, 221.1907, 203.1807
25	17.46	335.2232	381.2286	337.2384 [M + H]^+^	C_20_H_32_O_4_	319.2277, 301.2173, 289.2173, 273.2225, 255.2121, 221.1546, 207.1761, 199.1497, 193.1228, 175.1493
				354.2652 [M + H_2_O]^+^		
26	17.73	347.1849	393.1899	349.2000 [M + H]^+^	C_20_H_28_O_5_	303.1945, 285.1851, 274.2733, 240.2318, 221.1531, 207.1741, 175.1471
27	18.01	335.2225	381.2278	359.2196 [M + Na]^+^	C_20_H_32_O_4_	319.2268, 301.6121, 273.2212, 255.2112, 221.1540, 199.1494, 175.1490
				695.4498 [2M + Na]^+^		
28#	18.08 (acromatin C)	335.2226	381.2281	690.4946 [2M + H_2_O]^+^	C_20_H_32_O_4_	319.2274, 301.2170, 273.2220, 255.2115, 235.1695, 221.1543, 199.1485, 193.1228, 175.1493
29	18.35	335.2228	381.228	359.2162 [M + Na]^+^	C_20_H_32_O_4_	319.2274, 301.2170, 273.2220, 255.2115, 235.1698, 221.1543, 207.1731, 175.1493
30	18.65	---	---	345.2425 [M + Na]^+^ 667.4901 [2M + Na]^+^	C_20_H_34_O_3_	305.2476, 287.2370, 275.2368, 269.2266, 257.2272, 207.1748, 189.1643, 177.1639
31	18.79	---	---	662.5363 [2M + H_2_O]^+^	C_20_H_34_O_3_	305.2482, 287.2376, 275.2368, 269.2271,257.2288, 229.1962, 207.1753, 189.1646, 177.1646, 161.1337, 149.1328
32	18.84	335.2231	381.2287	359.2205 [M + Na]^+^ 690.4948 [2M + H_2_O]^+^ 695.4514 [2M + Na]^+^	C_20_H_32_O_4_	319.2279, 305.2486, 301.2169, 287.2375, 273.2223, 255.2117, 221.1548, 207.1755, 199.1490, 189.1647, 175.1492
33	19.20	349.2385	395.2438	368.2815 [M + H_2_O]^+^ 718.5308 [2M + H_2_O]^+^	C_21_H_34_O_4_	333.2448, 319.2286, 301.2179, 283.2076, 273.2235, 255.2127, 235.1706, 221.1554, 207.1403, 193.1242, 175.1493, 161.1339
34	19.31	349.2379	395.2432	---	C_21_H_34_O_4_	335.2238, 319.2288, 301.2180, 289.2183, 283.2069, 273.2222, 255.2126, 221.1554, 207.1403, 193.1242, 175.1493, 161.1339
35	19.45	335.2223	381.2276	359.2200 [M + Na]^+^ 673.4678 [2M + H]^+^	C_20_H_32_O_4_	319.2267, 301.2168, 291.2322, 273.2218, 255.2110, 221.1540, 207.1749, 203.1799, 189.1640, 175.1494
36#	19.52 (acromatin B)	---	---	345.2401 [M + Na]^+^ 667.4901 [2M + Na]^+^	C_20_H_34_O_3_	305.2476, 287.2370, 275.2368, 269.2266, 257.2271, 207.1748, 189.1643, 177.1639, 161.1328, 149.1325
37#	20.17 (acromatin A)	---	---	658.5023 [2M + H_2_O]^+^ 663.4627 [2M + Na]^+^	C_20_H_32_O_3_	303.2314, 285.2208, 275.2384, 257.2264, 205.1589, 201.1648, 187.1484, 177.1638, 149.1330
38	22.20	319.2278	365.2329	---	C_20_H_32_O_3_	303.2325, 285.2217, 257.2268, 233.1548, 221.1542, 201.1646, 175.1483, 163.1487, 147.1175

# Represented compounds that were identified with reference substances. * All compounds were detected using the dominant formate adduct ions [M + HCOO]^−^ and deprotonated molecule ions [M − H]^−^. ** the data obtained under positive mode offered more information on fragment ions and the positive mode was then chosen for structural elucidation. --- Represented no corresponded ions were observed.

## Supplementary Materials

The following are available online at https://www.mdpi.com/1420-3049/24/17/3073/s1, Figure S1: Chromatograms of *B. aromatica* extract with different extract method, Figure S2: Chromatograms of *B. aromatica* extract with different extract solvent, Figure S3: Chromatograms of *B. aromatica* extract with different extract time.

## Abbreviations

UHPLC	Ultra-high
QToF	Quadrupole Time of Flight
MDF	Mass defect filter
FSKD	forskolin-type diterpenoids
TCM	Traditional Chinese medicines
