# Supplementary material for: Rapid Screening of Forskolin-Type Diterpenoids of Blumea aromatica DC Using Ultra-High-Performance Liquid Chromatography Tandem Quadrupole Time-Of-Flight Mass Spectrometry Based on the Mass Defect Filtering Approach
Source: Molecules. 2019 Aug 23;24(17):3073. doi: 10.3390/molecules24173073 (PMC6749246; doi:10.3390/molecules24173073)
Supplement: Supplementary file 1 [file molecules-24-03073-s001.pdf]

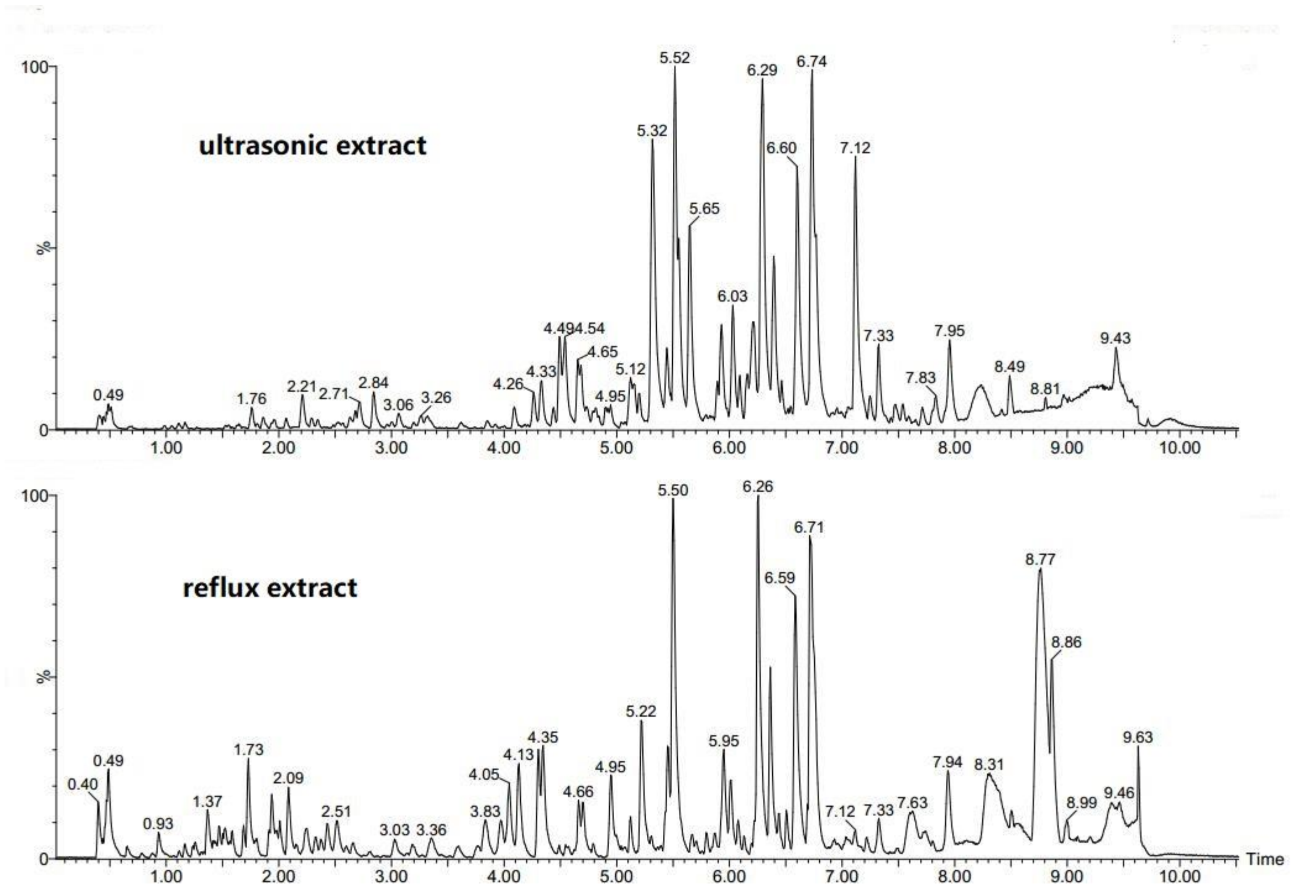


**Figure S1.** Chromatograms of *B. aromatica* extract with different extract method

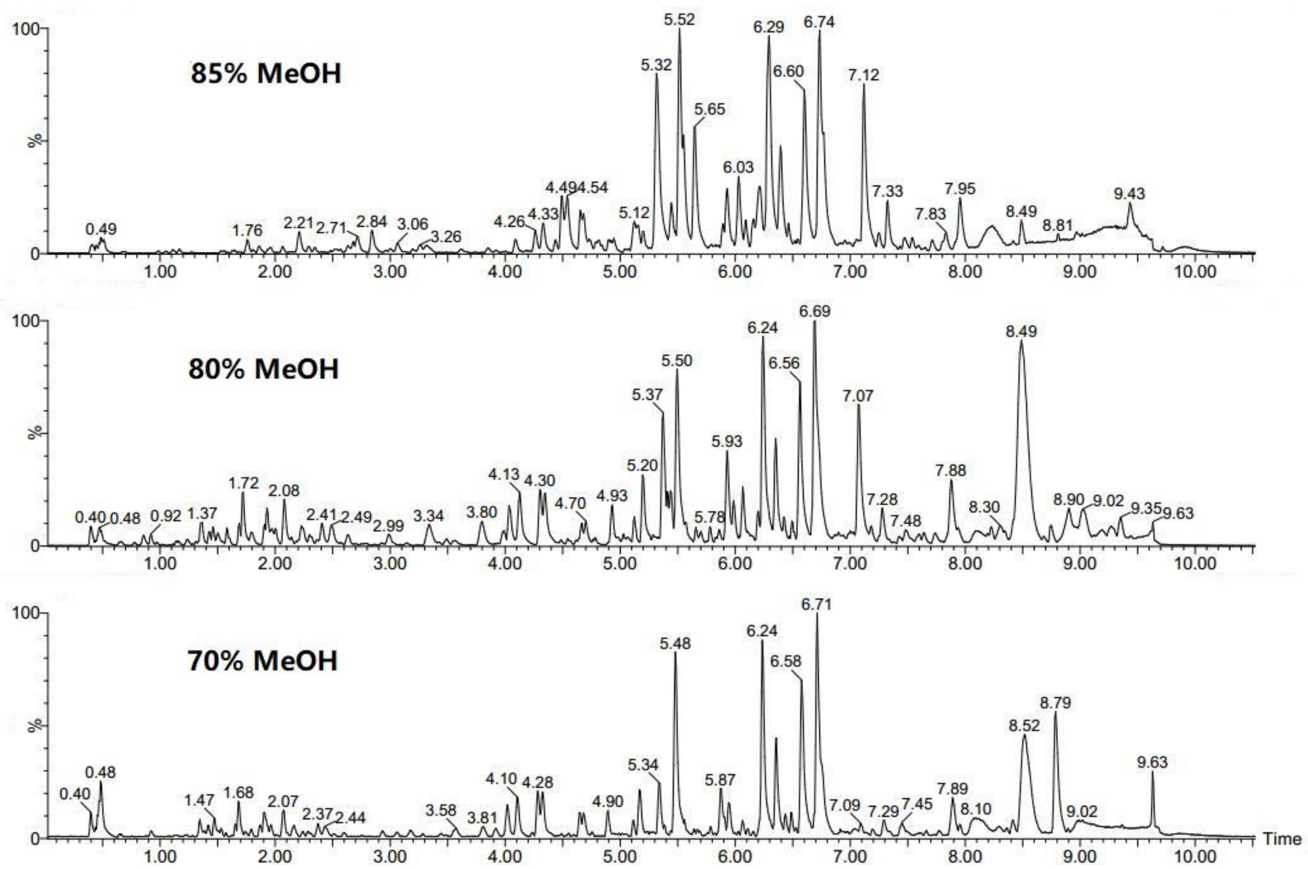


**Figure S2.** Chromatograms of *B. aromatica* extract with different extract solvent

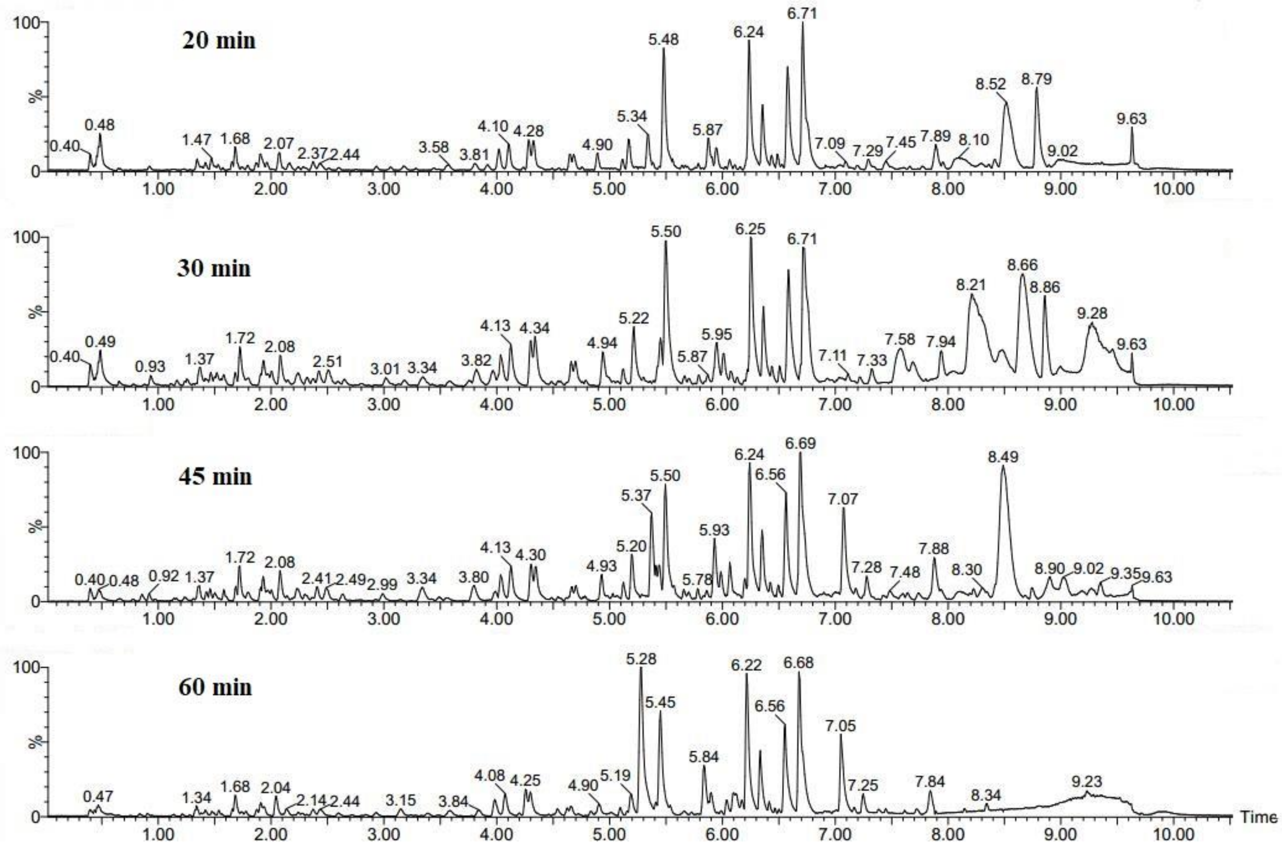


**Figure S3.** Chromatograms of *B. aromatica* extract with different extract time
